# Hydrogen-bonded network in the salt 4-methyl-1*H*-imidazol-3-ium picrate

**DOI:** 10.1107/S205698901600712X

**Published:** 2016-05-04

**Authors:** Xue-gang Song, Ping Su, Xing-man Xu

**Affiliations:** a College of Chemistry, Central China Normal University, Wuhan 430079, People’s Republic of China

**Keywords:** crystal structure, salt, 4-methyimidazole, picric acid, uninodal {4^2^.8^5^} net

## Abstract

In the title molecular salt, C_4_H_7_N_2_^+^·C_6_H_2_N_3_O_7_^−^, the phenolic proton of the starting picric acid has been transferred to the imidazole N atom. The nitro groups are twisted away from the benzene ring plane, making dihedral angles of 12.8 (2), 9.2 (4) and 29.3 (2)°. In the crystal, the component ions are linked into chains along [010] *via* N—H⋯O and bifurcated N—H⋯(O,O) hydrogen bonds. These chains are further linked by weak C—H⋯O hydrogen bonds into a three-dimensional network. The complex three-dimensional network can be topologically simplified into a 4-connected uninodal net with the point symbol {4.8^5^}.

## Abstract 2

A salt formed from 4-methyl­imidazole and picric acid was obtained in methanol solution. A three-dimensional hydrogen-bonded network is observed which can be topologically simplified into a uninodal 5-connected {4^2^.8^5^} net.

## Chemical context   

Co-crystallization, the crystallization of more than one solid component into a new compound, forming a new co-crystal or molecular salt, is a well known research field involving, for example, active pharmaceutical ingredients (Aitipamula *et al.*, 2015 ▸; Weyna *et al.*, 2012 ▸; Robinson, 2010 ▸; Arenas-García *et al.*, 2010 ▸) and crystal engineering (Manoj *et al.*, 2014 ▸). 4-Methyl­imidazole is an often used pharmaceutical inter­mediate (Shimpi *et al.*, 2014 ▸). The study of its crystallization can facilitate its related organic synthesis and theoretical optimization calculations. Picric acid, as a strong organic proton-donating reagent, is often adopted 2as an organic acid in the synthesis of co-crystallized complexes. Herein, we report the crystal structure of the molecular salt, 4-methyl­imidazolium picrate, (I). Future work will concentrate on how the crystallization behavior is affected by the solvent and temperature.
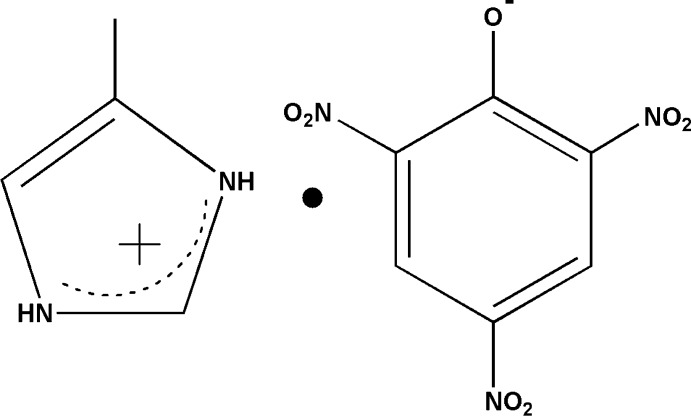


## Structural commentary   

**Figure 1:**
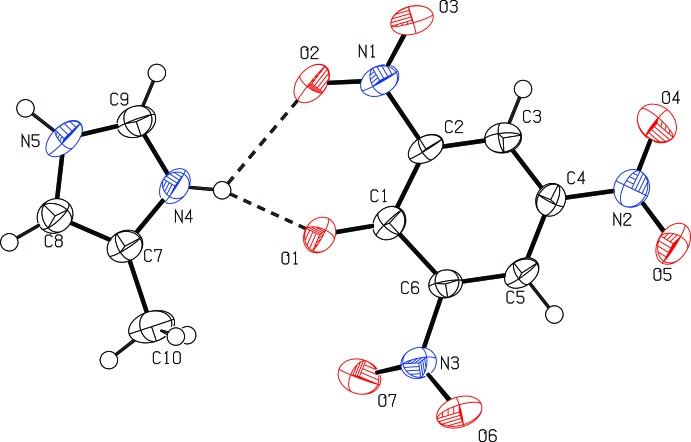
Mol­ecular structure of (I), showing the atom-numbering scheme. Displacement ellipsoids are drawn at the 50% probability level. Hydrogen bonds are shown as dashed lines.

The asymmetric unit of (I) consists of one 4-methyl­imidazolium cation and one picrate anion (Fig. 1 ▸). The phenolic proton in the original picric acid starting material was transferred from the picric acid OH group to the imidazole nitro­gen atom, forming a molecular salt. In the picrate anion, the C—O_phenol_ bond distance is shorter than in an earlier reported un-deprotonated compound [1.33 (2) Å; Bertolasi *et al.*, 2011 ▸] with a value of 1.244 (2) Å in (I). The adjacent C1—C2 [1.453 (2) Å] and C1—C6 [1.457 (3) Å] bonds are also lengthened from the values expected in a completely delocalized benzene ring. The C2—C1—C6 angle [111.0 (2)°] is smaller by *ca* 10° than the average value of the other five phenyl inner angles [121.8 (1)°]. This is mainly due to the electron-withdrawing effect of the three nitro groups attached to the aromatic π system, delocalizing electron density on the phenolate oxygen atom over the π system. The three nitro groups, N1/O2/O3, N2/O4/O5 and N3/O6/O6, are twisted away from the benzene ring plane, making dihedral angles of 12.8 (2), 9.2 (4) and 29.3 (2)°, respectively. In the 4-methyl­imidazolium cation, the C9—N4 [1.321 (3) Å] and C9—N5 [1.304 (3) Å] bond lengths are similar to each other due to the delocalizing effect; this is in contrast to the un-protonated 4-methylimidazole mol­ecule in the co-crystal of 8-hydroxyquinoline and 5-methyl-1*H*-imidazole [C—N = 1.305 (4) and 1.340 4 Å; Liu & Meng, 2006 ▸].

## Supra­molecular features   

**Figure 2:**
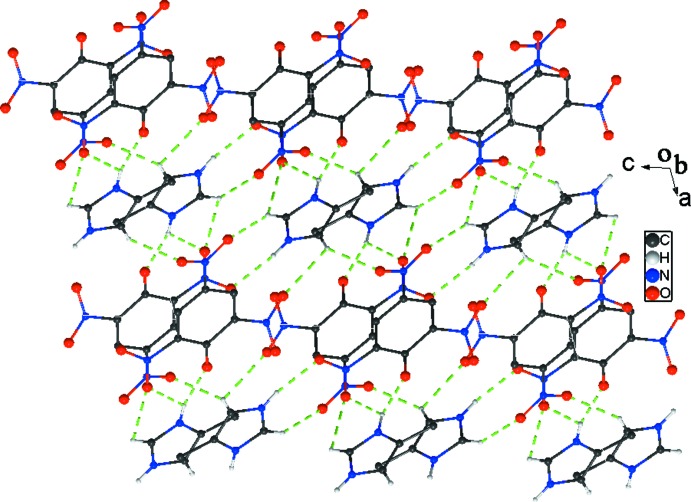
Part of the crystal structure of (I), showing the formation of the three-dimensional network. N—H⋯O Hydrogen bonds and C—H⋯O inter­actions are shown as green dashed lines. For the sake of clarity, H atoms not involved in the motif have been omitted.

In the crystal structure of (I), the component ions are linked into chains along [010] by N—H⋯O hydrogen bonds (Table 1 ▸, Fig. 2 ▸), one of which is bifurcated, N—H⋯(O,O). The chains are linked by C—H⋯O inter­actions, forming a three-dimensional framework. In the cation, all H atoms except for the methyl group H atoms act as hydrogen-bond donors. Each cation is bonded to four adjacent picrate anions. In turn, each picrate anion utilizes the one phenolic and four nitro oxygen atoms, acting as hydrogen-bond acceptors, linked to four 4-methyl­imidazolium cations. No other inter­actions such as π–π and C—H⋯π are observed (Spek, 2009 ▸).

**Figure 3:**
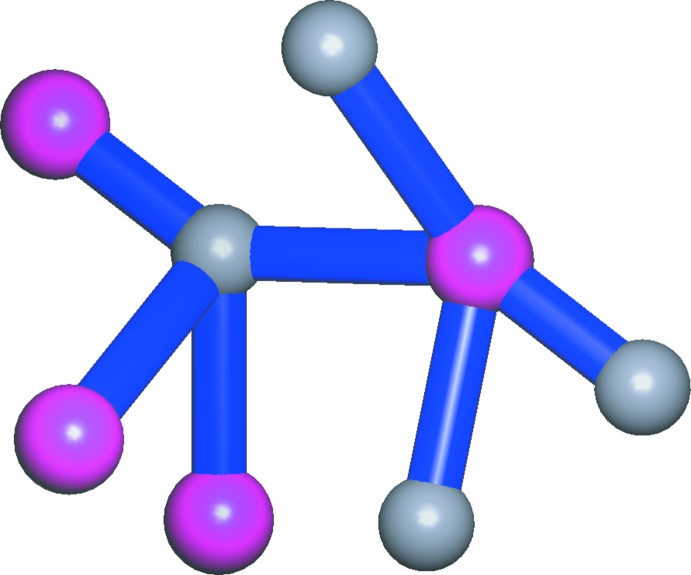
Part of the crystal structure of (I), showing the topologically connected relationship between 4-methyl­imidazolium and picrate ions (shown as gray and pink balls, respectively).

**Figure 4:**
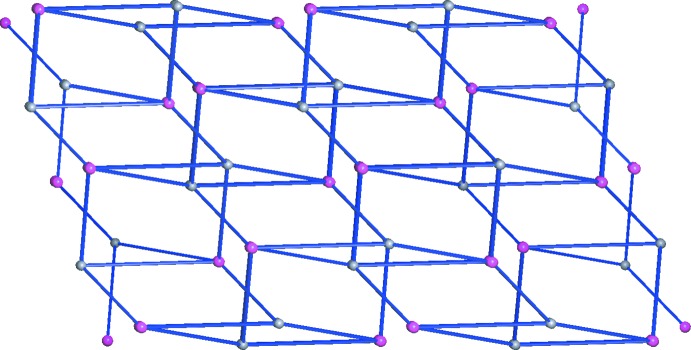
A schematic view of the formation of the 4-connected topological network in (I) when the cations and anions are regarded as four-connected nodes. The gray and pink spheres represent the 4-methyl­imidazolium cations and picrate anions, respectively.

In order to better understand the three-dimensional structure, we can regard both the cation and anion as 4-connected nodes (Fig. 3 ▸), *i.e*. each one 4-methyl­imidazolium ion links with four other picrate ions, and *vice versa*. Thus, the whole network is simplified into a uninodal 4-connected net with the point symbol {4.8^5^} (Baburin & Blatov, 2007 ▸; Blatov *et al.*, 2014 ▸) (Fig. 4 ▸).

## Database survey   

A CSD search (CSD Version 5.37 plus one update; Groom *et al.*, 2016 ▸) found some analogs of the title compound, *viz*. BEZGEU (2-methylimidazolium picrate 2-methylimidazole; Dhanabal *et al.*, 2013 ▸) and QAKYOS (2-methyl-1*H*-imidazol-3-ium 2,4,6-trinitrophenolate; Dutkiewicz *et al.*, 2011 ▸). A structural comparison indicates that the two nitro­gen atoms are preferably hydrogen-bonded to the picrate anions, of which one is bifurcated and the other is linear.

## Synthesis and crystallization   

Equivalent molar amounts of 4-methyl imidazole (1.0 mmol, 80.0 mg) and picric acid (1 mmol, 230.0mg) were dissolved in 95% methanol (40.0 ml). The mixture was stirred for half an hour at room temperature and then filtered. The resulting yellow solution was kept in air for two weeks. Needle-shaped yellow crystals of (I) suitable for single-crystal X-ray diffraction analysis were grown at the bottom of the vessel by slow evaporation of the solution. The crystals were separated by filtration (yield, 75%, *ca* 0.23 g).

## Refinement   

Crystal data, data collection and structure refinement details are summarized in Table 2 ▸. H atoms bonded to C atoms were positioned geometrically with C—H = 0.93 Å (aromatic) or 0.96 Å (meth­yl) and refined using a riding model [*U*_iso_(H) = 1.2*U*_eq_(Caromatic) or 1.5*U*_eq_(Cmeth­yl)]. H atoms bonded to N atoms were found in Fourier difference maps; N—H distances were refined freely with *U*_iso_(H) = 1.2*U*_eq_(N).

## Supplementary Material

Crystal structure: contains datablock(s) global, I. DOI: 10.1107/S205698901600712X/lh5809sup1.cif

Structure factors: contains datablock(s) I. DOI: 10.1107/S205698901600712X/lh5809Isup2.hkl

Supporting information file. DOI: 10.1107/S205698901600712X/lh5809Isup3.cml

CCDC reference: 1476728

Additional supporting information:  crystallographic information; 3D view; checkCIF report

## Figures and Tables

**Table 1:** Hydrogen-bond geometry (Å, °)

*D*—H⋯*A*	*D*—H	H⋯*A*	*D*⋯*A*	*D*—H⋯*A*
N4—H4*A*⋯O2	0.84 (2)	2.46 (2)	3.013 (3)	124 (2)
N4—H4*A*⋯O1	0.84 (2)	1.88 (2)	2.687 (2)	160 (2)
N5—H5*A*⋯O3^i^	0.87 (2)	2.07 (3)	2.898 (3)	160 (2)
C8—H8⋯O4^ii^	0.93	2.50	3.302 (3)	145
C9—H9⋯O5^iii^	0.93	2.39	3.242 (3)	152

Symmetry codes: (i) 
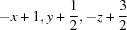
; (ii) 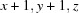; (iii) 
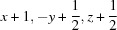
.

**Table 2:** Experimental details

Crystal data	
Chemical formula	C_4_H_7_N_2_^+^·C_6_H_2_N_3_O_7_^−^
*M*_r_	311.22
Crystal system, space group	Monoclinic, *P*2_1_/*c*
Temperature (K)	298
*a*, *b*, *c* (Å)	9.3079 (17), 9.4339 (17), 15.195 (3)
β (°)	107.835 (2)
*V* (Å^3^)	1270.2 (4)
*Z*	4
Radiation type	Mo *K*α
μ (mm^−1^)	0.14
Crystal size (mm)	0.30 × 0.05 × 0.02

Data collection	
Diffractometer	Bruker *SMART* CCD
Absorption correction	Multi-scan (*SADABS*; Sheldrick, 2008 ▸)
*T*_min_, *T*_max_	0.936, 0.992
No. of measured, independent and observed [*I* > 2σ(*I*)] reflections	12952, 2489, 1539
*R*_int_	0.142
(sin θ/λ)_max_ (Å^−1^)	0.616

Refinement	
*R*[*F* ^2^ > 2σ(*F* ^2^)], *wR*(*F* ^2^), *S*	0.052, 0.110, 1.04
No. of reflections	2489
No. of parameters	206
H-atom treatment	H atoms treated by a mixture of independent and constrained refinement
Δρ_max_, Δρ_min_ (e Å^−3^)	0.25, −0.22

Computer programs: *SMART* and *SAINT-Plus* (Bruker, 2001 ▸), *SHELXS* and *SHELXTL* (Sheldrick, 2008 ▸), *SHELXL2014* (Sheldrick, 2015 ▸) and *DIAMOND* (Brandenburg, 2006 ▸).

## supplementary crystallographic information

### Crystal data

**Table:**

C_4_H_7_N_2_^+^·C_6_H_2_N_3_O_7_^−^	*F*(000) = 640
*M**_r_* = 311.22	*D*_x_ = 1.628 Mg m^−^^3^
Monoclinic, *P*2_1_/*c*	Mo *K*α radiation, λ = 0.71073 Å
*a* = 9.3079 (17) Å	Cell parameters from 1754 reflections
*b* = 9.4339 (17) Å	θ = 2.3–22.1°
*c* = 15.195 (3) Å	µ = 0.14 mm^−^^1^
β = 107.835 (2)°	*T* = 298 K
*V* = 1270.2 (4) Å^3^	Needle, yellow
*Z* = 4	0.30 × 0.05 × 0.02 mm

### Data collection

**Table:**

Bruker SMART CCD diffractometer	1539 reflections with *I* > 2σ(*I*)
φ and ω scans	*R*_int_ = 0.142
Absorption correction: multi-scan (*SADABS*; Sheldrick, 2008)	θ_max_ = 26.0°, θ_min_ = 2.3°
*T*_min_ = 0.936, *T*_max_ = 0.992	*h* = −11→11
12952 measured reflections	*k* = −11→11
2489 independent reflections	*l* = −18→18

### Refinement

**Table:**

Refinement on *F*^2^	0 restraints
Least-squares matrix: full	Hydrogen site location: inferred from neighbouring sites
*R*[*F*^2^ > 2σ(*F*^2^)] = 0.052	H atoms treated by a mixture of independent and constrained refinement
*wR*(*F*^2^) = 0.110	*w* = 1/[σ^2^(*F*_o_^2^) + (0.0154*P*)^2^] where *P* = (*F*_o_^2^ + 2*F*_c_^2^)/3
*S* = 1.04	(Δ/σ)_max_ < 0.001
2489 reflections	Δρ_max_ = 0.25 e Å^−^^3^
206 parameters	Δρ_min_ = −0.22 e Å^−^^3^

### Special details

**Table:**

Geometry. All esds (except the esd in the dihedral angle between two l.s. planes) are estimated using the full covariance matrix. The cell esds are taken into account individually in the estimation of esds in distances, angles and torsion angles; correlations between esds in cell parameters are only used when they are defined by crystal symmetry. An approximate (isotropic) treatment of cell esds is used for estimating esds involving l.s. planes.

### Fractional atomic coordinates and isotropic or equivalent isotropic displacement parameters (Å^2^)

**Table:**

	*x*	*y*	*z*	*U*_iso_*/*U*_eq_	
C1	0.1017 (2)	0.4075 (2)	0.43900 (15)	0.0339 (6)	
C2	0.0815 (2)	0.3083 (2)	0.50742 (14)	0.0340 (6)	
C3	−0.0320 (2)	0.2096 (2)	0.49046 (15)	0.0350 (6)	
H3	−0.0386	0.1486	0.5371	0.042*	
C4	−0.1356 (2)	0.2015 (2)	0.40413 (15)	0.0318 (5)	
C5	−0.1265 (2)	0.2912 (2)	0.33388 (15)	0.0344 (6)	
H5	−0.1983	0.2860	0.2759	0.041*	
C6	−0.0126 (2)	0.3863 (2)	0.34989 (14)	0.0326 (6)	
C7	0.4896 (2)	0.7421 (2)	0.51495 (15)	0.0367 (6)	
C8	0.6087 (3)	0.8040 (3)	0.57572 (16)	0.0420 (6)	
H8	0.6633	0.8808	0.5646	0.050*	
C9	0.5366 (3)	0.6298 (3)	0.64773 (16)	0.0438 (7)	
H9	0.5314	0.5658	0.6932	0.053*	
C10	0.4074 (3)	0.7745 (3)	0.41634 (16)	0.0552 (7)	
H10A	0.3094	0.8119	0.4116	0.083*	
H10B	0.3966	0.6894	0.3803	0.083*	
H10C	0.4634	0.8432	0.3936	0.083*	
N1	0.1822 (2)	0.3139 (2)	0.60151 (13)	0.0421 (5)	
N2	−0.2537 (2)	0.0971 (2)	0.38594 (15)	0.0414 (5)	
N3	−0.0051 (3)	0.4734 (2)	0.27120 (13)	0.0438 (5)	
N4	0.4464 (2)	0.6333 (2)	0.56162 (13)	0.0390 (5)	
H4A	0.369 (3)	0.584 (3)	0.5399 (16)	0.047*	
N5	0.6343 (2)	0.7322 (2)	0.65746 (14)	0.0450 (6)	
H5A	0.706 (3)	0.757 (3)	0.7063 (18)	0.054*	
O1	0.19859 (18)	0.50281 (17)	0.45332 (11)	0.0487 (5)	
O2	0.2988 (2)	0.3814 (2)	0.62018 (11)	0.0623 (6)	
O3	0.1436 (2)	0.2490 (2)	0.66125 (12)	0.0682 (6)	
O4	−0.2498 (2)	0.00651 (19)	0.44540 (13)	0.0600 (5)	
O5	−0.35582 (18)	0.10262 (18)	0.31206 (12)	0.0520 (5)	
O6	−0.1232 (2)	0.4962 (2)	0.21012 (13)	0.0744 (6)	
O7	0.1172 (2)	0.5136 (2)	0.26798 (12)	0.0673 (6)	

### Atomic displacement parameters (Å^2^)

**Table:**

	*U*^11^	*U*^22^	*U*^33^	*U*^12^	*U*^13^	*U*^23^
C1	0.0342 (14)	0.0300 (14)	0.0336 (13)	0.0044 (11)	0.0044 (11)	−0.0040 (11)
C2	0.0361 (13)	0.0374 (14)	0.0223 (12)	0.0054 (11)	0.0001 (10)	−0.0027 (10)
C3	0.0416 (14)	0.0332 (13)	0.0295 (14)	0.0067 (11)	0.0100 (11)	0.0010 (11)
C4	0.0311 (13)	0.0310 (13)	0.0318 (13)	−0.0012 (11)	0.0074 (11)	−0.0043 (11)
C5	0.0373 (13)	0.0358 (13)	0.0245 (12)	0.0039 (11)	0.0010 (10)	−0.0043 (11)
C6	0.0389 (14)	0.0296 (13)	0.0262 (12)	0.0024 (11)	0.0055 (10)	0.0018 (10)
C7	0.0410 (15)	0.0341 (14)	0.0313 (14)	0.0045 (11)	0.0056 (11)	−0.0018 (11)
C8	0.0456 (15)	0.0401 (15)	0.0371 (15)	−0.0006 (12)	0.0080 (12)	−0.0018 (12)
C9	0.0514 (17)	0.0405 (15)	0.0329 (15)	0.0040 (13)	0.0030 (13)	0.0019 (11)
C10	0.0690 (18)	0.0555 (18)	0.0323 (15)	0.0077 (15)	0.0023 (13)	0.0037 (13)
N1	0.0473 (13)	0.0444 (14)	0.0281 (12)	0.0037 (11)	0.0020 (10)	−0.0017 (10)
N2	0.0427 (13)	0.0394 (13)	0.0421 (13)	0.0010 (10)	0.0131 (11)	−0.0065 (11)
N3	0.0503 (14)	0.0377 (13)	0.0349 (12)	−0.0030 (11)	0.0007 (11)	0.0058 (10)
N4	0.0366 (12)	0.0352 (12)	0.0353 (12)	−0.0032 (10)	−0.0033 (10)	−0.0046 (9)
N5	0.0415 (13)	0.0515 (14)	0.0306 (12)	0.0014 (11)	−0.0058 (10)	−0.0104 (11)
O1	0.0515 (11)	0.0442 (11)	0.0398 (10)	−0.0130 (9)	−0.0017 (8)	−0.0003 (8)
O2	0.0481 (11)	0.0838 (16)	0.0408 (11)	−0.0196 (11)	−0.0074 (9)	0.0005 (10)
O3	0.0797 (14)	0.0853 (15)	0.0274 (10)	−0.0219 (12)	−0.0016 (10)	0.0122 (10)
O4	0.0664 (13)	0.0493 (12)	0.0609 (13)	−0.0107 (10)	0.0144 (10)	0.0134 (10)
O5	0.0459 (11)	0.0590 (13)	0.0428 (11)	−0.0098 (9)	0.0014 (9)	−0.0118 (9)
O6	0.0702 (14)	0.0825 (15)	0.0472 (12)	−0.0069 (11)	−0.0163 (10)	0.0287 (11)
O7	0.0618 (13)	0.0821 (15)	0.0546 (12)	−0.0132 (12)	0.0126 (10)	0.0241 (11)

### Geometric parameters (Å, º)

**Table:**

C1—O1	1.244 (2)	C8—H8	0.9300
C1—C2	1.453 (3)	C9—N5	1.304 (3)
C1—C6	1.457 (3)	C9—N4	1.321 (3)
C2—C3	1.372 (3)	C9—H9	0.9300
C2—N1	1.451 (3)	C10—H10A	0.9600
C3—C4	1.372 (3)	C10—H10B	0.9600
C3—H3	0.9300	C10—H10C	0.9600
C4—C5	1.385 (3)	N1—O2	1.214 (2)
C4—N2	1.438 (3)	N1—O3	1.236 (2)
C5—C6	1.353 (3)	N2—O5	1.230 (2)
C5—H5	0.9300	N2—O4	1.236 (2)
C6—N3	1.470 (3)	N3—O7	1.215 (2)
C7—C8	1.340 (3)	N3—O6	1.222 (2)
C7—N4	1.375 (3)	N4—H4A	0.84 (2)
C7—C10	1.491 (3)	N5—H5A	0.87 (2)
C8—N5	1.370 (3)		
			
O1—C1—C2	125.9 (2)	N5—C9—N4	107.6 (2)
O1—C1—C6	123.1 (2)	N5—C9—H9	126.2
C2—C1—C6	111.0 (2)	N4—C9—H9	126.2
C3—C2—N1	116.0 (2)	C7—C10—H10A	109.5
C3—C2—C1	124.3 (2)	C7—C10—H10B	109.5
N1—C2—C1	119.7 (2)	H10A—C10—H10B	109.5
C4—C3—C2	119.5 (2)	C7—C10—H10C	109.5
C4—C3—H3	120.2	H10A—C10—H10C	109.5
C2—C3—H3	120.2	H10B—C10—H10C	109.5
C3—C4—C5	120.8 (2)	O2—N1—O3	121.9 (2)
C3—C4—N2	119.6 (2)	O2—N1—C2	120.7 (2)
C5—C4—N2	119.6 (2)	O3—N1—C2	117.4 (2)
C6—C5—C4	119.8 (2)	O5—N2—O4	122.5 (2)
C6—C5—H5	120.1	O5—N2—C4	118.5 (2)
C4—C5—H5	120.1	O4—N2—C4	118.9 (2)
C5—C6—C1	124.5 (2)	O7—N3—O6	123.5 (2)
C5—C6—N3	117.02 (19)	O7—N3—C6	119.07 (19)
C1—C6—N3	118.4 (2)	O6—N3—C6	117.4 (2)
C8—C7—N4	106.3 (2)	C9—N4—C7	109.4 (2)
C8—C7—C10	131.6 (2)	C9—N4—H4A	125.9 (17)
N4—C7—C10	122.1 (2)	C7—N4—H4A	124.4 (17)
C7—C8—N5	106.6 (2)	C9—N5—C8	110.0 (2)
C7—C8—H8	126.7	C9—N5—H5A	128.9 (17)
N5—C8—H8	126.7	C8—N5—H5A	121.1 (17)

### Hydrogen-bond geometry (Å, º)

**Table:**

*D*—H···*A*	*D*—H	H···*A*	*D*···*A*	*D*—H···*A*
N4—H4*A*···O2	0.84 (2)	2.46 (2)	3.013 (3)	124 (2)
N4—H4*A*···O1	0.84 (2)	1.88 (2)	2.687 (2)	160 (2)
N5—H5*A*···O3^i^	0.87 (2)	2.07 (3)	2.898 (3)	160 (2)
C8—H8···O4^ii^	0.93	2.50	3.302 (3)	145
C9—H9···O5^iii^	0.93	2.39	3.242 (3)	152

Symmetry codes: (i) −*x*+1, *y*+1/2, −*z*+3/2; (ii) *x*+1, *y*+1, *z*; (iii) *x*+1, −*y*+1/2, *z*+1/2.

## References

[bb1] Aitipamula, S., Mapp, L. K., Wong, A. B. H., Chow, P. S. & Tan, R. B. H. (2015). *CrystEngComm*, **17**, 9323–9335.

[bb2] Arenas-García, J. I., Herrera-Ruiz, D., Mondragón-Vásquez, K., Morales-Rojas, H. & Höpfl, H. (2010). *Cryst. Growth Des.* **10**, 3732–3742.

[bb3] Baburin, I. A. & Blatov, V. A. (2007). *Acta Cryst.* B**63**, 791–802. 10.1107/S0108768107033137 17873448

[bb4] Bertolasi, V., Gilli, P. & Gilli, G. (2011). *Cryst. Growth Des.* **11**, 2724–2735.

[bb5] Blatov, V. A., Shevchenko, A. P. & Proserpio, D. M. (2014). *Cryst. Growth Des.* **14**, 3576–3586.

[bb6] Brandenburg, K. (2006). *DIAMOND*. Crystal Impact GbR, Bonn, Germany.

[bb7] Bruker (2001). *SAINT-Plus* and *SMART*. Bruker AXS Inc., Madison, Wisconsin, USA.

[bb8] Dhanabal, T., Sethuram, M., Amirthaganesan, G. & Das, S. K. (2013). *J. Mol. Struct.* **1045**, 112–123.

[bb9] Dutkiewicz, G., Samshuddin, S., Narayana, B., Yathirajan, H. S. & Kubicki, M. (2011). *Acta Cryst.* E**67**, o235. 10.1107/S1600536810053390 PMC3050384 21522735

[bb10] Groom, C. R., Bruno, I. J., Lightfoot, M. P. & Ward, S. C. (2016). *Acta Cryst* B**72**, 171–179. 10.1107/S2052520616003954 PMC4822653 27048719

[bb11] Liu, Z.-X. & Meng, X.-G. (2006). *Acta Cryst.* E**62**, o1286–o1288.

[bb12] Manoj, K., Tamura, R., Takahashi, H. & Tsue, H. (2014). *CrystEngComm*, **16**, 5811–5819.

[bb13] Robinson, D. I. (2010). *Org. Process Res. Dev.* **14**, 946–959.

[bb14] Sheldrick, G. M. (2008). *Acta Cryst.* A**64**, 112–122. 10.1107/S0108767307043930 18156677

[bb15] Sheldrick, G. M. (2015). *Acta Cryst.* C**71**, 3–8.

[bb18] Shimpi, M. R. , Childs, S. L., Boström, D., & Velaga, S. P. (2014). *CrystEngComm*, **16**, 8984–8993.

[bb16] Spek, A. L. (2009). *Acta Cryst.* D**65**, 148–155. 10.1107/S090744490804362X PMC2631630 19171970

[bb17] Weyna, D. R., Cheney, M. L., Shan, N., Hanna, M., Wojtas, Ł. & Zaworotko, M. J. (2012). *CrystEngComm*, **14**, 2377–2380.

